# Evaluation and Analysis of Eco-Security in Environmentally Sensitive Areas Using an Emergy Ecological Footprint

**DOI:** 10.3390/ijerph14020136

**Published:** 2017-01-30

**Authors:** Han-Shen Chen

**Affiliations:** 1 School of Health Diet and Industry Management, Chung Shan Medical University, No.110, Sec. 1, Jianguo N. Rd., Taichung City 40201, Taiwan; allen9750@yahoo.com.tw; Tel.: +886-4-2473-0022; 2 Department of Medical Management, Chung Shan Medical University Hospital, No.110, Sec. 1, Jianguo N. Rd., Taichung City 40201, Taiwan

**Keywords:** eco-security, land use and cover change (LUCC), sustainability development and assessment

## Abstract

In this paper, the overall ecological and environmental sustainability in the Cing-Jing region in Taiwan is examined. As land use and cover change has been found to be an important analysis method, an emergy ecological footprint model was applied and the eco-security assessed to ensure authorities maintain a balance between ecological preservation and tourism development. While the ecological environment in the Cing-Jing region from 2008 to 2014 was found to be within safe levels, all related indices had increased considerably. A Grey model was used to predict the 2015–2024 ecological carrying capacities, from which it was found that there is expected to be a large increase in per capita ecological footprints (EFs), meaning that in the future there is going to be a larger ecological deficit and a higher ecological pressure index (EFI), with the eco-security predicted to reach a Grade 2 intermediate level in 2022. As the Cing-Jing region is predicted to become ecologically unsustainable, local, regional, and national governments need to implement regulations to strictly control the land use in the Cing-Jing region. This study demonstrated that emergy EF (EEF) theory application can give objective guidance to decision-makers to ensure that recreational non-urban eco-security can be maintained at a safe level.

## 1. Introduction

Due to urbanization and agricultural and tourism land use, there has been increasing research interest in the effects of global land use and land cover [1,2,3,4]. The International Geosphere-Biosphere Programme (IGBP) and the International Human Dimensions Programme on Global Environmental Change (IHDP) have jointly promoted land use and cover change (LUCC) and the Global Land Project (GLP). The impact of urban expansion on natural environments has also become a major focus for the Scientific Committee on Problems of the Environment (SCOPE) and, as a result of these rising global concerns, the Urban Peri-Urban Environmental Change (PU-ECH) Research Project was activated. Global and regional scale LUCC research studies and temporal and spatial scales have generally been focused on administrative areas, vulnerable areas, and other ecologically sensitive areas, and current research has been divided into administrative regions, such as continents, countries, provinces, cities, and counties [5,6], and natural regions, such as environmentally sensitive areas and coastal areas [7,8]. 

Previous studies on land use and the associated environmental impacts have tended to only focus on exploring the effects from a single perspective, but there have been few studies that have investigated the impact of land development and land use changes on the natural environment or have decomposed, analyzed, and synthesized the effect of complex socio-economic variables on natural ecosystems and their interaction with land use to determine land-use change and spatial pattern trends [9,10,11]. To date, econometric models [12,13], statistical models [4,14,15,16,17,18], cellular automata models [1], and integrated models [19] have been the most commonly used models, and in recent years, to examine land-use changes and spatial pattern trends, models have been developed that integrate the interactions between the complex natural ecosystem and the socioeconomic processes [2,20,21].

Research has focused on land use change processes, change modes, the driving forces of the LUCC mechanism, and LUCC environmental effects, and regional land use change models have been developed in an attempt to understand the possible correlations between regional land development, natural resources, and human activities, and to investigate the conditions under different situations to predict future land use change trends. Studies have also included the effect of LUCC on soil, on the possibility of natural disasters, and on resource use impact. Comprehensive evaluations have been conducted on ecosystem services, and ecological evaluation methods have been developed to evaluate ecological LUCC impacts on ecosystems [22,23,24].

As non-urban land has a wide range of uses such as food supply, climate regulation, water resources maintenance, biological diversity support, and human recreation, to develop sustainable management strategies, it is necessary to understand the environmental conservation issues facing these uses. Many past studies have examined land use in relation to disaster prevention [25,26] and developed associated management-oriented mitigation plans. [27,28]. Other more logistical studies have focused more on environmental carrying capacity (ECC) evaluation methods and have developed quantitative analytical methods for direct and indirect measurements [29,30]. However, few of these studies have explored dynamic development trends. Therefore, the objective of this study is to explore the land use change process and the impact this has had on the environment.

Global climate change has already had noticeable effects around the world such as desertification, reduced ecosystem resilience, and loss of biodiversity [24]. As far back as 1972, the United Nations Declaration on the Human Environment and Eco-security, in addition to outlining principles for sustainable human development projects, raised concerns about the preservation of food sources and ecosystems, providing a new perspective for the assessment of environmental resources, sustainable development, and human survival [23]. In line with these changes, in 1989, the International Institute for Applied Systems Analysis (IIASA) developed the eco-security concept that encompassed human life, health, happiness, fundamental rights, livelihood security sources, necessary resources, social order, and the human capacity to adapt to environmental changes and other climate threats [29]. As a result, the focus on ecological problems became more prominent, with many research institutions and scholars developing a range of different indicators and measurement system models to evaluate eco-security on different regional scales [31] using eco-security analyses, forecasting, and eco-security early warning [30].

Recent analyses of these measurement systems and models, however, have identified several major deficiencies [20,23,25,27,28,29,30]. One of the main problems has been the over-complicated and difficult to assess eco-security indicators, with very few actually able to dynamically predict sustainable development changes. In addition, because no complete evaluation index system has been developed to accompany the models, many of the indicators are difficult to quantify and consequently have poor operability [29]. Finally, some of the evaluation index measurement models required information that made them impractical [31].

Therefore, even though concerns about environmental security have been expressed for more than 40 years, eco-security evaluation research using quantitative evaluation methods and criteria is still at the exploratory stage because of changes in ecosystem services due to land use changes. Ecosystem services estimate the economic value of a system in terms of its ability to provide societal services, which can increase or decrease depending on changes to the overall eco-security situation [32]. 

While regional land use change has been a major research area in the past decade, this study is the first to apply an emergy ecological footprint (EEF) model to understand the changes in land-use parameters. The focus area for this study was the Cing-Jing region in Taiwan. For the analysis, the LUCC and EEF models were combined, and the ecosystem elements of the ecological tracing model were reclassified based on the various land-use purposes in the Cing-Jing region. The EEF model was then applied to analyze the temporal changes in terms of the ecosystem service needs and the ecological carrying capacity or the ecosystem services supplied. From this analysis, an eco-security evaluation system was derived for the Cing-Jing region to predict future needs in terms of spatial planning, land management, and resource conservation policy development. 

The remainder of this paper is organized as follows. In Section 2, the Cing-Jing study area is described, after which the EEF method is applied to analyze the land use changes and the Ecological Footprint Method used to measure the ecological resources in the region to determine the carrying capacity. The corresponding ecological quotiety is then used to assess the relationship between development in the Cing-Jing region and the ecological environment. Using data from 2008 to 2014, the per capita ECC and per capita EEF are calculated to examine the land use changes over time. In Section 3, the results are presented and Grey theory is applied to predict the 2015–2024 per capita ecological footprint (EF) and per capita EC in the Cing-Jing region. Section 4 presents the discussion in which the key problems and possible solutions are examined. Section 5 gives the conclusion and the strengths of the methodology.

## 2. Materials and Methods

### 2.1. Study Area

The Cing-Jing region research site used in this study is located in Central Taiwan and is a popular location for leisure and recreation attractions. Because of its distinctive terrain and the unique cultural heritage of the indigenous Truku people, the Cing-Jing region is seen as an important asset for the development of international tourism. However, except for those built in early stages of development, such as the Cing-Jing Guest Hotel and the cabins and camping sites already planned for, there is no available land in the region to build additional accommodation facilities because of land use restrictions. Therefore, to meet tourist demand, local residents provide bed and breakfast (B&B) type facilities for local and international visitors. However, because this mountain area has deep terrain and fragile geology as well as heavy and concentrated rainfall, the construction of new tourist facilities could severely impact the ecological environment and the water and land resource maintenance. Therefore, to ensure that the tourist industry can develop sustainably, the ecological systems are maintained, and to reduce costs and increase operational efficiency, there is an urgent need for a sustainable development assessment of the tourist and recreational facilities to guide decision-makers on effective resource use. 

The evaluation model developed in this paper provides a valuable assessment of the biocapacity in the Cing-Jing region, as well as identifying any discrepancies in the model’s application. Therefore, this case study provides some guidance for the development of sustainable resource management models in similar geographical environments.

### 2.2. Method

#### 2.2.1. Land Use Change Patterns

The EF concept has become more accepted in the past few years as a viable method for measuring sustainability. However, most of the analytical methods that have been developed have tended to examine the biologically productive land necessary to support human activities and the consequent waste production. In particular, the energy conversion concept has been the primary method used to examine EFs. More recently, an improved method based on emergy analysis has been proposed to determine EFs, the EEF [32,33,34], in which the EA is integrated into the EF framework to calculate the EFs and the ecological carrying capacity. In this model, the natural resources, the various types of human consumption, and the energy flow levels are converted into a solar emergy value based on emergy transformity, after which emergy density is used to convert the solar emergy of each consumption item for each corresponding biologically productive area so that the EF and ecological carrying capacity can be determined. These results are then used to identify the conditions necessary to ensure sustainable development. Haberl et al. (2001) [35] observed that the success of the EEF in measuring and determining sustainability hinged on the quantitative indices used to represent the EFs and ecological carrying capacity. 

Therefore, in this study, the EEF method was applied to measure the land-use change in the Cing-Jing region. The EEF, which incorporates emergy theory, focuses on the consumption in six biologically productive land types (fossil energy land, arable land, grassland, forests, built-up land, and water sources) as well as the resources provided by each. The analysis uses common energy value metrics, which allows for the energy value density, or the number of all renewable energy sources per unit area, to be determined, with the results being easy to understand by regional decision-makers.

The EEF is divided into biological resources and energy [33,34]. Biological resources refer to the agricultural products, forest products, livestock products, and aquatic products, respectively produced from the farmland, woodland, grasslands, and water bodies, and energy refers to the energy used for this production such as coal, crude oil, natural gas, or electric power.

To determine the per capita ecological footprint (*ef*), the biological productivity of the land types, the physical quantity corresponding to the energy values, and the (*c_i_*) available per capita consumption are divided by (*P_1_*), as shown in Equation (1):

(1) $$ef=\sum_{i=1}^{n}a_{i}=\sum_{i=1}^{n}c_{i}/P_{1}$$

where *i* is the resource consumption type; *ef* is per capita EEF; *a_i_* is the per capita consumption of resources in the *i*-th area; *c_i_* is the *i*-th value per capita energy resource consumption measured in solar energy joules (sej); and *P_1_* is the regional energy density value where *P_1_* = the area total energy value/regional area, in sej/m^2^.

The ecological carrying capacity (ECC) measures all renewable energy resources in the study region and the global average emergy density ratio value. To determine the regional ECC energy value, first, the renewable per capita resources in the region such as solar, wind, rain, potential chemical energy, as well as the energy value of the rotation of the Earth, were calculated. To avoid double counting, the maximum value was taken as the regional value, and the available resources per capita energy supply (*e*) were divided by the energy density value (*P_2_*) to determine the per capita energy value for the ecological carrying capacity (*ec*), as shown in Equation (2):

(2) $$ec=e/P_{2}$$

where *ec* is the per capita emergy ecological carrying capacity; *e* is the supply of energy resources per capita; and *P_2_* is the global energy density in *P_1_* units, which was calculated as follows: *P_2_* = the total energy value of the Earth/Earth area = Earth’s biosphere major energy values, the reference value for which was 1.583 × 1025 sej/a [36].

When the ECC of a region is less than that demanded by the EF, an ecological deficit (ED) occurs, indicating that the ecological footprint of the region exceeds its ecological carrying capacity and the corresponding development model is not sustainable. When the ECC is greater than that required by the EF standards, an ecological remainder (ER) occurs, indicating that the ecological capacity of the region is sufficient and that the development model is sustainable. Rees [37] reported that an ED is usually a result of humans placing excessive demands on the ecosystem; therefore, to maintain sustainable ecological development, ecological demand must be reduced.

Moore et al. [38] adopted the EF to examine ED/ER in Vancouver, with the results indicating a severe deficit. As EEF calculations are based on official regional, national, and international government department statistics, accuracy is important for determining the ECC. Therefore, in this paper, specific indicators were developed to accurately assess the ECC, and they are as follows.

#### 2.2.2. Ecological Pressure Index (EFI)

In this study, the Ecological Footprint Method was used to measure the ecological resources in the Cing-Jing region to determine whether the carrying capacity of the land in the area was being exceeded. EFI is defined as the renewable resources per capita ecological footprint (*ef'*) divided by the emergy ecological carrying capacity ratio (*ec*) as shown in Equation (3):

EFI = *ef'*/*ec* (3)

where EFI is the ecological pressure index; *ef'* is the renewable resources per capita Ecological Footprint; and *ec* is the per capita emergy ecological carrying capacity.

EFI involves comparing the resource and energy expenditures with the ecological capacity in a region to evaluate resource utilization and determine whether the resource and environmental conditions have sustainable development characteristics. When the EFI = 1, the energy ecological carrying capacity supply and demand are balanced and the ecosystems are in a critical sustainable development state. When the EFI < 1, the emergy ecological carrying capacity is greater than the ecological footprint and the ecosystem load is normal, so it is being sustainably developed. When the EFI > 1, the ecological footprint is greater than the emergy ecological carrying capacity, meaning that human consumption has exceeded the ecological carrying capacity and the area is being unsustainably developed.

#### 2.2.3. Corresponding Ecological Quotiety

To compensate for the ecological deficit, the corresponding ecological quotiety (DS) [39,40,41,42] was used to measure the functional relationship between Cing-Jing regional development and the ecological environment, as defined in Equation (4):

(4) $$\mathrm{DS}=\frac{ef+ec}{\sqrt{ef^{2}+ec^{2}}}$$

where DS is the corresponding regional per capita ecological quotiety; *ef* is the per capita EEF; and *ec* is the per capita ecological carrying capacity.

The DS is an important ecological harmony indicator for the ecological sustainability state in the Cing-Jing area. The results showed that, when the ecological pressure was lower, the ecological environment was better coordinated, and when the ecological pressure was large, the ecological environment was less coordinated. The DS can be used to determine the ecological pressure index threshold, which illuminates the ecological security level and the ecological security warning level.

When the DS is closer to 1, the Cing-Jing area EEF is higher than the ecological carrying capacity, indicating a worsening of the ecological harmony and a lower degree of regional development. Conversely, when the DS is closer to 0, the Cing-Jing area EEF and ecological capacity balance are well coordinated; therefore, the corresponding ecological quotiety shows the extent of the eco-security in the region.

Per capita EC and per capita EF were used to calculate the 2008–2014 EFIs of the research area (Table 1). It was found that the EFI increased from 1.08 in 2008 to 2.14 in 2014. From 2008 to 2014, the DS in the Cing-Jing area was gradually approaching 1; therefore, although the Cing-Jing area in 2008–2014 was in a relatively safe ecological state, the ecological pressure index showed that the area had been under increasing pressure for the previous seven years, gradually stressing the ecosystem and threatening the overall eco-security.

## 3. Results

Grey theory, developed originally by Deng [43], is a multidisciplinary, generic theory that deals with systems characterized by a lack of comprehensive information. Grey theory includes systems analysis, data processing, modeling, prediction, decision-making, and control, and Grey prediction models have been used in many applications [44,45,46]. In contrast to statistical methods, the original series in the time series grey model, called the GM (1,1) mode, has greater potency. Based on the fundamental grey prediction model data, by examining the EF in this time series we are able to provide a comparison for per capita EEF or ECC in different years, so as to predict how the resource environment in Cing-Jing is going to develop in the future. The 2015–2024 per capita EEF and per capita ECC in the Cing-Jing region were calculated (Table 2). Although the 2015–2020 per capita ECCs in the Cing-Jing region increased slightly, the 2020–2024 capacities decreased. In addition, the 2015–2024 per capita EEFs were found to grow substantially, which is expected to enlarge the regional per capita ecological deficit and render the entire region’s development unsustainable, thereby further endangering the ecological environment. 

Based on the prediction results, the 2015–2024 per capita ecological deficit and surplus values were determined (Table 2). The results showed that from 2015 to 2024 ecological deficits in the Cing-Jing region are expected to gradually increase. Because the EF and per capita demand is predicted to increase annually and the natural supplies predicted to decrease, the per capita ecological deficit in 2024 in the Cing-Jing region is anticipated to be −23.03 hm^2^/cap, indicating that human demand is going to considerably exceed the load capacity of the natural systems, causing significant ecological and environmental deterioration. From 2015 to 2024, even though the eco-security level of the Cing-Jing region is predicted to satisfy the safety standard, because the EFI is predicted to increase, there will be a rapid deterioration in the ecological environment. 

Using the EFI-related data and integrating these data with the eco-security standards, the predicted eco-security from 2015 to 2024 was determined (Table 2). In 2018, the Cing-Jing region is expected to reach a Grade 2 intermediate level, with the early-warning level expected to increase rapidly. This may be a direct result of the predicted increase in the tourist demand for resources and services (e.g., bus service, road constructions, and waste production). Therefore, it is suggested that the Cing-Jing regional management continue to track demand as well as develop sustainability incentives. As can be seen, these EEF calculation methods can give a precise evaluation of the tourist load capacity in the Cing-Jing region and inform decision-makers regarding the issues that need to be resolved.

## 4. Discussion

This study applied the EEF method and adopted a comprehensive evaluation method made up of the EFI and the grey prediction model to evaluate and predict the eco-security in the Cing-Jing region, for which the following conclusions were made.

The results showed that the Cing-Jing region EFI increased from 2008 to 2014, indicating that the overall situation of the Cing-Jing region ecosystem was not improving and that there was significant ecological pressure [32]; however, in the same period, the Cing-Jing region eco-security was still within a safe level [31]. Nonetheless, by 2014, the Cing-Jing region had several major problems; the vegetation was becoming severely degraded, there were fewer bird varieties, and water pollution was increasing. These results were primarily due to the fact that several relationships had not been sufficiently dealt with; for example, the relationship between mountain slope resource protection and rational development and utilization, and the relationships between current interests, long-term interests, and overall interests. Generally, however, the results demonstrated that authorities had focused on ecological security in the Cing-Jing region and implemented certain improvement measures. 

The EEF model was used to calculate the ecological deficit (ED) and the ecological remainder (ER). The EFIs were determined through a comprehensive analysis of the 2008–2014 eco-security, and it was found that, from 2008 to 2014, the Cing-Jing region ecological environment was in a safe state; however, the related indices increased annually.

The grey model was adopted to predict the 2015–2024 EEFs and ecological carrying capacities in the Cing-Jing region, after which the ecological deficit values and EFIs were calculated to predict the eco-security trends and early warning status. The predictions found that, from 2015 to 2024, ecological carrying capacities in the Cing-Jing region are expected to decrease, the regional per capita EF is expected to significantly increase, and the ecological deficit and EFI are also expected to increase. The eco-security evaluation prediction indicated, however, that, from 2015 to 2024, the eco-security level in the Cing-Jing region is expected to stay at a safe level; however, when combined with the EFIs, the eco-security status is expected to decline, meaning that the overall ecological environment is expected to deteriorate. The eco-security level from 2015 to 2024 is predicted to be greater than the previous seven years and is anticipated to reach a Grade 2 intermediate level by 2022. The early warning level is also predicted to increase rapidly.

Based on the above prediction results, to effectively control biocapacity and to achieve the conservation and sustainable development goals in the Cing-Jing region, land-use regulations need to continue to be applied and a more explicit conservation program focused on Cing-Jing’s regional resource characteristics developed to ensure long-term preservation of the unique and scenic natural environments, biodiversity, and historical relics.

The tourism gains in the Cing-Jing region can only be sustained if the tourism providers, the central government, and local authorities work together to address the problems that are hindering sustainable tourism development. To this end, tourism suppliers (e.g., services, transportation, food and beverage, and accommodation) can contribute by maximizing local labor employment; however, local authorities need to determine tourism development’s long-term scope and identify an appropriate carrying capacity congruent with the tourist destination. As local cultures and native lifestyles need to be preserved, sustainability initiatives need to be developed by the local community as they are the beneficiaries of such development.

The National Scenic Area Administration management office should nominate specific personnel to provide for patrol and inspection services so as to maintain strict control of recreational activities in the park and prevent any tourist behavior that may damage or contaminate the environment. As well as meeting the demand for transport and recreation by implementing appropriate sustainable transport policies, it is also suggested that CO_2_ emissions reduction and energy-saving leisure tourism be popularized. 

Past research has explored theoretical hypotheses, basic concepts, calculation methods, empirical applications, and deficiencies related to the EF model in Taiwan [29,31,32,47] and developed several EF theories and calculation methods on different levels. Lee and Peng [48] extended previous studies by analyzing Taiwan’s EF from 2008 to 2011. Lee [49] used a time-series analysis to explore the land footprint, the carbon footprint, and the water footprint from 2000 to 2011 in Taiwan. The findings indicate that the land footprint in Taiwan was 5.39 gha in 2000 and reduced to 3.63 gha in 2011. The research in this paper revealed that the EF and the ED in Cing-Jing have been increasing annually, indicating that local resources are being overused and tremendous pressure is being placed on the environment. Therefore, it is necessary for the national and local governments to act immediately to reduce the ED. The Taiwanese government has been promoting an energy tax or carbon tax, (also known as the “green tax”), which has prompted many people to buy low-polluting motorcycles because of the discounts that have been introduced for more environmentally friendly engines. There are several limitations to the study in this paper. First, the environmental impact of tourists can vary in space and in time as tourist activity is influenced by such factors as climate, holidays, and celebratory events. The frequency of tourist activities and the concentration of tourists can change the ecosystem and cause permanent damage at certain times of the year through concentrated excessive pollution emissions. However, as the EEF is calculated on an annual basis, these impact variations were not considered. 

Second, because of the data access and inspection research limitations, only seven years of pooled time series analysis information was available. As this time scale was not large enough, it was not possible to fully explain the change process for every explanatory variable in the time-series, which influenced the explanatory strength of the model. Further, as this is a newly established indicator system, there is a lack of comparative data from other areas or countries. Therefore, to fully analyze the usefulness of the model and the indicators developed in this paper, further studies need to be conducted in similar areas around the world.

## 5. Conclusions

In this paper, the Cing-Jing region in Taiwan was examined to assess its overall ecological and environmental sustainability. As LUCC has been found to be an important method for the investigation of the causes of global environment change, the EEF model was applied to construct a land-use change model to assess past and predict future sustainable development trends. 

The eco-security of the Cing-Jing region was also assessed to give guidance to responsible agencies to ensure a balance between ecological preservation and tourism development. It was found that, while the ecological environment of the Cing-Jing region from 2008 to 2014 were within safe levels, all related indices had increased considerably. A Grey model was used to predict the 2015–2024 ecological carrying capacities, from which it was found that there is expected to be a large increase in per capita EFs, meaning that in the future there is going to be a larger ecological deficit and a higher EFI. The eco-security from 2015 to 2024 was also higher and is predicted to reach a Grade 2 intermediate level in 2022. Therefore, over time, as the Cing-Jing region is predicted to become ecologically unsustainable, local, regional, and national governments need to implement regulations to strictly control the land use in the Cing-Jing region. 

This study has shown that developing appropriate measurement indices and using EEF theory to quantitatively analyze mountainside recreational areas to evaluate ecological security can give solid objective guidance to decision-makers as to the types of measures that need to be taken to ensure that eco-security in recreational non-urban areas can be maintained at a safe level.

## Figures and Tables

**Table 1:** Cing-Jing region 2008–2014 ecological pressure index (EFI), ecological quotiety (DS), and eco-security (ES).

Year	2008	2009	2010	2011	2012	2013	2014
EFI	1.08	1.34	1.36	1.43	1.80	1.97	2.14
DS	1.92	1.76	1.68	1.52	1.47	1.25	1.13
ES level	2	2	2	2	2	2	2
ES evaluation	Safe	Safe	Safe	Safe	Safe	Safe	Safe

**Table 2:** ES evaluation and early warning stage.

Year	Per Capita ECC	Per Capita EEF	Ecological Deficit/Surplus	EFI			ES Early Warning State	
				Index	Grade	Safety Status	Level	Status
2015	3.92	9.24	−5.32	2.36	2	Safe	1	Mild
2016	3.95	10.40	−6.45	2.63	2	Safe	1	Mild
2017	3.99	11.71	−7.72	2.93	2	Safe	1	Mild
2018	4.03	13.18	−9.15	3.27	2	Safe	1	Mild
2019	4.07	14.83	−10.76	3.64	2	Safe	1	Mild
2020	4.02	16.70	−12.68	4.15	2	Safe	1	Mild
2021	3.96	18.79	−14.83	4.74	2	Safe	1	Mild
2022	3.93	21.15	−17.22	5.38	2	Safe	2	Inter-mediate
2023	3.87	23.80	−19.93	6.15	2	Safe	2	Inter-mediate
2024	3.76	26.79	−23.03	7.13	2	Safe	2	Inter-mediate

Source: Data collected and organized by this study.
